# Effects of endothelial nitric oxide synthase on mouse arteriovenous fistula hemodynamics

**DOI:** 10.1038/s41598-023-49573-5

**Published:** 2023-12-20

**Authors:** Shelly Baltazar, Hannah Northrup, Joshua Chang, Maheshika Somarathna, Tatyana Isayeva Waldrop, Timmy Lee, Yan-Ting Shiu

**Affiliations:** 1 Department of Biomedical Engineering, University of Utah, Salt Lake City, UT USA; 2 Division of Nephrology and Hypertension, Department of Internal Medicine, University of Utah, 30 N Mario Capecchi Drive, 3rd Floor South, Salt Lake City, UT 84112 USA; 3 Division of Nephrology, Department of Medicine, University of Alabama at Birmingham, Birmingham, AL USA; 4 Veterans Affairs Medical Center, Birmingham, AL USA; 5 Veterans Affairs Medical Center, Salt Lake City, UT USA

**Keywords:** Biomedical engineering, Nephrology

## Abstract

Newly created arteriovenous fistulas (AVFs) often fail to mature for dialysis use due to disturbed blood flow at and near the AVF anastomosis. The disturbed flow inhibits the endothelial nitric oxide synthase (NOS3) pathway, thus decreasing the production of nitric oxide, a vasodilator. Previously, our group reported that NOS3 expression levels affect AVF lumen size in a mouse model. In this study, we performed MRI-based computational fluid dynamics simulations to investigate the hemodynamical parameters (velocity, wall shear stress (WSS), and vorticity) in a mouse AVF model at day 7 and day 21 post-AVF creation using three NOS3 strains: overexpression (OE), knockout (KO), and wild-type (WT) control. This study is the first to reveal hemodynamics over time in mouse AVFs, consider spatial heterogeneity along the vein, and reveal the effect of NOS3 on the natural history of mouse AVF hemodynamics. From day 7 to day 21, OE has smoother streamlines and had significantly lower vorticity and WSS than WT and KO, suggesting that WSS was attempting to return to pre-surgery baseline, respectively. Our results conclude that the overexpression of NOS3 leads to desired optimal hemodynamics during AVF remodeling. Future studies can investigate enhancing the NOS3 pathway to improve AVF development.

## Introduction

Hemodialysis is the most common form of renal replacement therapy for end-stage kidney disease patients in the US and developed countries^1^. Before patients can start hemodialysis treatment, they must have a reliable vascular access point capable of supplying high flow rates to and from the dialysis machine^2,3^. Currently, the preferred vascular access for hemodialysis is the arteriovenous fistula (AVF) due to its higher long-term patency rates than other types of vascular access^4^. The AVF is created by surgically connecting an artery to a vein, usually in the upper extremity. After the creation of an AVF, its venous lumen must enlarge/dilate sufficiently to allow a high blood flow rate for hemodialysis; this is called AVF maturation. However, AVFs frequently fail to mature^2,4,5^. Factors contributing to maturation failure include, but are not limited to, the lack of arterial and venous dilation, early thrombosis, and venous stenosis due to the development of neointimal hyperplasia (NH)^3,6^. Still, the underlying mechanisms leading to these factors are not yet fully understood, and currently, no therapies are proven to enhance AVF maturation effectively.

Nitric oxide (NO) has been known to play an essential role in vascular health by regulating vascular homeostasis and its potent effect on vasodilation^7–9^. A critical cellular source of NO in the vasculature is the endothelial cells via the endothelial nitric oxide synthase (NOS3) pathway. Impairment of the NOS3 pathway has been shown to contribute to cardiovascular disorders, including atherosclerosis^9^. NO is synthesized from l-arginine in a reaction catalyzed by NOS3, a mechanosensitive enzyme activated by laminar blood flow over endothelial cells^9–11^. However, the blood flow in an AVF at and near the anastomosis is usually disturbed, nonuniform, and irregular^12^. Disturbed blood flow inhibits the activation of the NOS3 pathway in endothelial cells and thus decreases NO production^11^. Manipulating the NOS3 pathway through genetic modifications can aid in understanding the effect of NOS3 on the AVF remodeling process, which has yet to be fully characterized.

Our group has reported some aspects of AVF remodeling using three mouse strains with different NOS3 expression levels: overexpression (OE), knockout (KO), and wild-type (WT)^13,14^. Our geometric study found that the overexpression of NOS3 increased the AVF lumen size without affecting its shape, such as the anastomosis angle and tortuosity^14^. Our fluid–structure interaction (FSI) study, using a small cohort (n = 1 per strain) and a single time point (day 21 after AVF creation), found lower wall shear stress (WSS) in the NOS3 OE mouse AVF when compared to WT and NOS3 KO, with a focus on the AVF vein WSS averaged over 7 mm starting from the anastomosis, suggesting that WSS in NOS3 OE was attempting to return to pre-surgery baseline, a desired hemodynamic adaptation^13^. The FSI study also found that WSS results by FSI (which assumes a deformable vascular wall) and computational fluid dynamics (CFD) (which assumes a rigid vascular wall) are similar^13^. CFD has the advantage of reduced computational time. This study aims to investigate the effect of NOS3 on the hemodynamical parameters (velocity, WSS, and vorticity) of a carotid jugular mouse AVF model with three mouse strains (WT, NOS3 KO, and NOS3 OE) at day 7 and day 21 post-AVF creation, providing a larger sample size, an additional time point relevant to early remodeling, and a more detailed hemodynamic analysis in both the venous and arterial limbs of the AVF, with a more granular analysis within the venous limb, than previous work.

## Methods

### Mouse model and MRI acquisition

C57BL/6 WT mice and NOS3 KO mice on a C57BL/6 background were obtained from Jackson Laboratories (Bar Harbor, ME). NOS3 OE mice were provided by Dr. Christopher Kevil. Western blot and cyclic guanosine monophosphate levels confirmed the varying NOS3 expression levels in the three mouse strains before AVF creation^13^. Carotid-jugular AVFs were surgically created in young (3–4 month old) male mice by one surgeon, as previously described^15^.

Magnetic resonance image (MRI) acquisition was performed on mice 7 and 21 days post-AVF creation using procedures we have previously described^15^. Briefly, a two-dimensional, T2-weighted fast spin echo sequence with black-blood double inversion MRI was done on the mice to visualize the blood vessel lumen. There were six conditions in total: three mouse strains (WT, KO, and OE) at two time points (day 7 and day 21 post-AVF creation)^14^. The number of mice used for each condition was five for WT at day 7, three for WT at day 21, five for KO at day 7, three for KO at day 21, three for OE at day 7, and three for OE at day 21.

All animal studies and experiments were approved by the University of Alabama at Birmingham (UAB) Institutional Animal Care and Use Committee and in accordance with the National Institutes of Health guidelines. All methods are reported in accordance with ARRIVE guidelines, and animals were sacrificed appropriately, with isoflurane as the anesthesia agent and exsanguination under anesthesia as the euthanasia method.

### Mouse AVF lumen segmentation, reconstruction, and meshing

**Figure 1:**
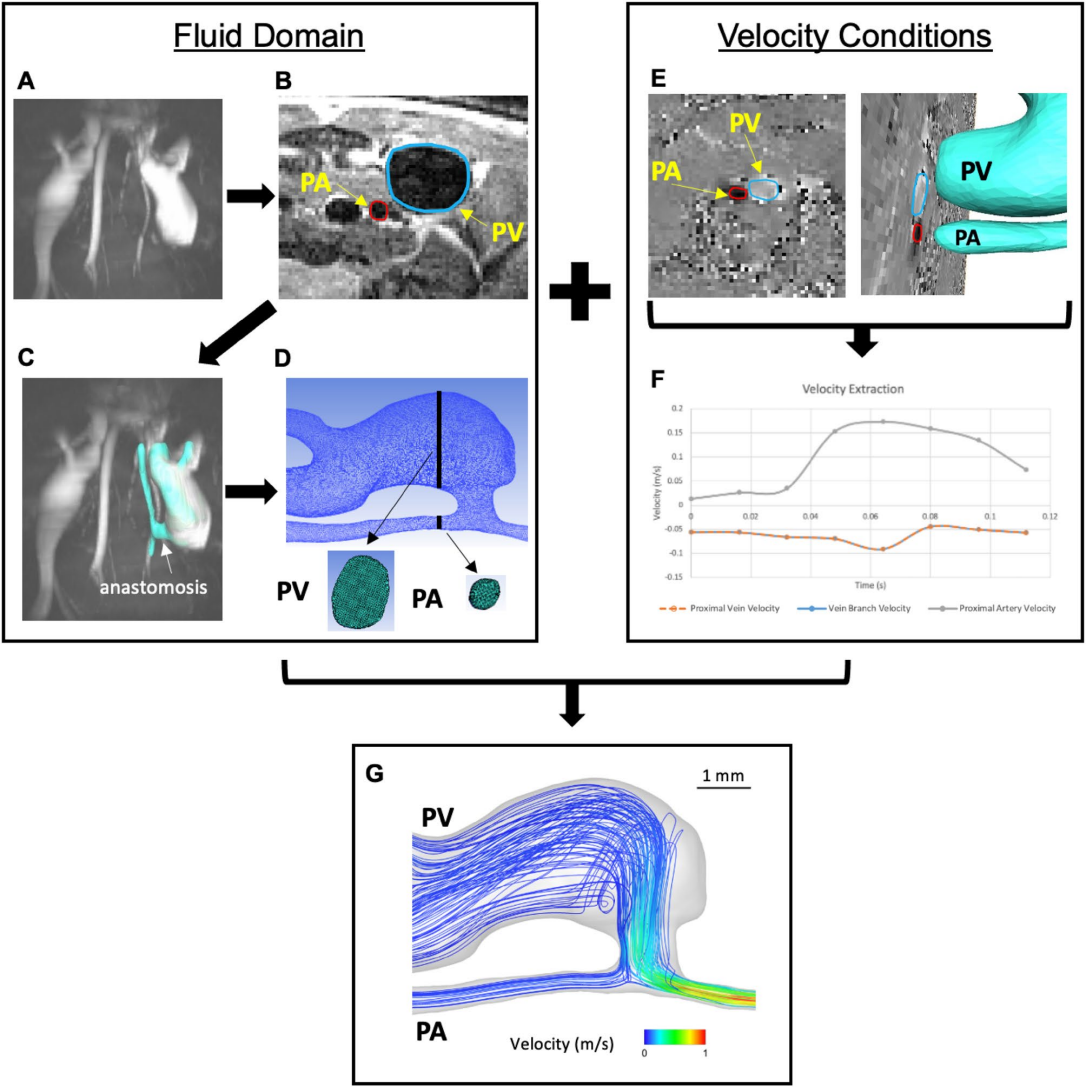
Computational fluid dynamic modeling pipeline. Fluid domain: (**A**) time-of-flight (TOF) MR images of the mouse AVF. (**B**) segmentation of the lumen of the proximal artery (PA) and proximal vein (PV). (**C**) 3D AVF reconstruction generated from the segmentation was aligned with the TOF images. (**D**) Tetrahedral volumetric mesh was created. Velocity conditions: (**E**) velocity was extracted from cine-phase MR images of the PA and PV over a cardiac cycle. (**F**) Graph of the PA and PV blood velocity over a cardiac cycle. (**G**) Simulation was performed using the fluid domain and velocity conditions.

All 22 mice in the experimental setting were used for the computational study. Three-dimensional reconstructions of the mice’s AVF lumen were generated in Amira 3D 2021.1 (Thermo Fisher Scientific, Waltham, Mass) from black-blood MRI scans^15^. Figure 1 shows the process of creating the fluid domain needed for CFD. Briefly, the AVF lumen in the black-blood MRI scans was segmented manually using the blowout tool. Then, the 2D sections were utilized to generate a 3D surface reconstruction in STL format, which was smoothed at 60 iterations with a lambda of 0.6 in Amira. The exported STL surface was then used in VMTK 1.4.0 (available at: www.vmtk.org) to add flow extensions to prevent entrance effects in the region of interest^16^. Using the extended 3D AVF lumen geometry in the STL file format, a high-resolution tetrahedral volumetric mesh was created in ANSYS ICEM CFD 2020 R2 (Ansys, Inc., Canonsburg, PA) with 1–1.5 million tetrahedral elements in the lumen domain with four prism layers at the wall, based on the previous mesh studies published by our group^15^. The tetrahedral elements are technically cells for solving the differential equations necessary to conduct CFD.

### Velocity extraction

The AVF blood flow velocity was extracted in ImageJ 1.53a (https://imagej.nih.gov/ij/) using 2D gradient echo velocity mapping scans (cine phase-contrast MRI). The blood flow velocity was extracted at 2 locations near the fistula: the proximal artery and the proximal vein (Fig. 1). Since the blood flow velocity was measured after the proximal vein and vein branch split, the blood flow velocity at the vein branch was assumed to be the same as the proximal vein.

### CFD modeling

The CFD simulation was performed in ANSYS Fluent 2020 R2 (Ansys, Inc., Canonsburg, PA) using the 3D volumetric meshes created in *“Mouse AVF Lumen Segmentation, Reconstruction, and Meshing”*^15^. The simulation domain included the proximal artery, distal artery, proximal vein, and vein branch. The inflow boundary condition (proximal artery) and two outflow boundary conditions (proximal vein and vein branch) were prescribed as pulsatile cross-sectional blood flow velocity extracted from the cine phase-contrast MRI^15^. The remaining outflow boundary condition (distal artery) was set as a pressure outlet with 0 gauge pressure. Simulation settings were previously described^15^. In brief, no-slip boundary conditions were prescribed at the vessel wall, and the vessel walls were assumed to be rigid. The blood was assumed to be incompressible and Newtonian with a density of 1050 kg/m^3^ and a dynamic blood viscosity of 0.0035 Pa s. The simulation ran for three cardiac cycles with 1280 time-steps over the cardiac cycle length (128 ms for a step size of 0.1 ms). The convergence criteria were x-, y-, and z-residuals of 1 × 10^–5^ and a total residual of 1 × 10^–5^^15^. Mesh and time-step size independence tests of this mouse AVF model were conducted by our previously published studies^15^.

### Post-Processing for hemodynamic parameters

Hemodynamic parameter results were calculated and extracted in Tecplot 360 EX 2019 R1 (Tecplot, Inc., Bellevue, WA), as previously described^15^. The hemodynamic parameters of interest in this study were velocity magnitude (Eq. 1), WSS magnitude (Eq. 2), and vorticity (Eq. 3). In all equations, u = blood velocity vector, x, y, z = coordinates, $\tau$ = shear stress, and w = wall. These parameters were averaged in Tecplot 360 over the third cardiac cycle.

1 $$u={({u}_{x}^{2}+{u}_{y}^{2}+{u}_{z}^{2})}^\frac{1}{2}$$

2 $$WSS={({\tau }_{w,x}^{2}+{\tau }_{w,y}^{2}+{\tau }_{w,z}^{2})}^\frac{1}{2}$$

3 $$Vorticity= \nabla \times u$$

For each animal in all groups, VMTK was used to calculate the centerline and centerline normal values on the 3D surface reconstructions with points at 0.1 mm intervals from the anastomosis for both the proximal vein and proximal artery. The calculated centerline normal values were created in MATLAB (MATLAB, Natick, MA) and were imported with the centerline values into Tecplot to generate slices normal to the centerline at 0.1 mm intervals^16^. The hemodynamic parameters were extracted 4 mm from the anastomosis into the AVF vein (before the vein branch bifurcation) and 4 mm from the anastomosis into the proximal artery. The resulting data was then analyzed in GraphPad Prism 9.5.1 (GraphPad Software, San Diego, CA). For each NOS3 strain and time point, each of the three hemodynamic parameters was averaged for each mouse along the 4-mm segment, then averaged within each group. This resulted in data for three hemodynamic parameter results for 6 conditions^16^.

### MRI and CFD derived velocity comparison

Velocity was extracted from the cine-phase MRI using the method described in *“Velocity Extraction”*. The CFD-derived velocity was extracted in Tecplot by taking a slice in the exact same location the cine-phase MRI was taken on the mouse AVF to measure the velocity. This slice was used to extract the velocity over one cardiac cycle. The averaged CFD-derived and MRI velocities over a cardiac cycle were compared, and an agreement plot was created in GraphPad Prism (Supplementary Figs. S1 and S2).

### Statistical analysis

All statistical analysis and graphs were done and created in GraphPad Prism. Shapiro–Wilk normality tests were done to test the data for a normal distribution. To test for significance, when the data had a normal distribution, an unpaired parametric t-test was conducted. When the data did not have a normal distribution, an unpaired nonparametric Mann–Whitney test was performed.

## Results

### Qualitative AVF hemodynamic parameters

**Figure 2:**
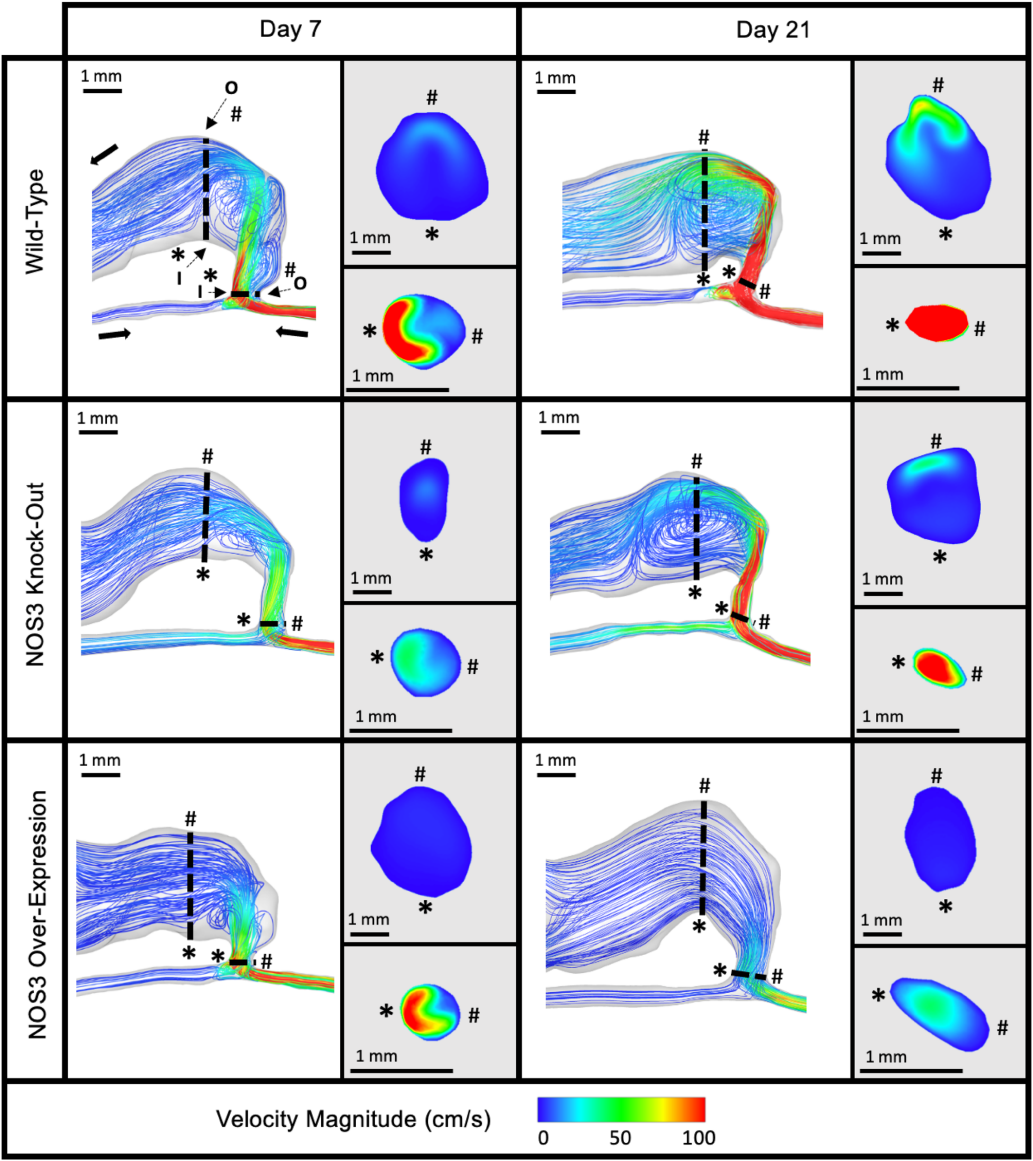
Representative velocity streamlines in mouse AVF lumen. Representative velocity streamline color plot for the three mouse NOS3 strains at 7 and 21 days post-AVF creation at systole. Slices of AVF lumen velocity at the anastomosis (bottom) and 3 mm into the AVF venous limb (top) are shown next to each plot showing the whole AVF. The velocity color bar applies to all mouse AVFs. The red represents velocity at and above 100 cm/s.

**Figure 3:**
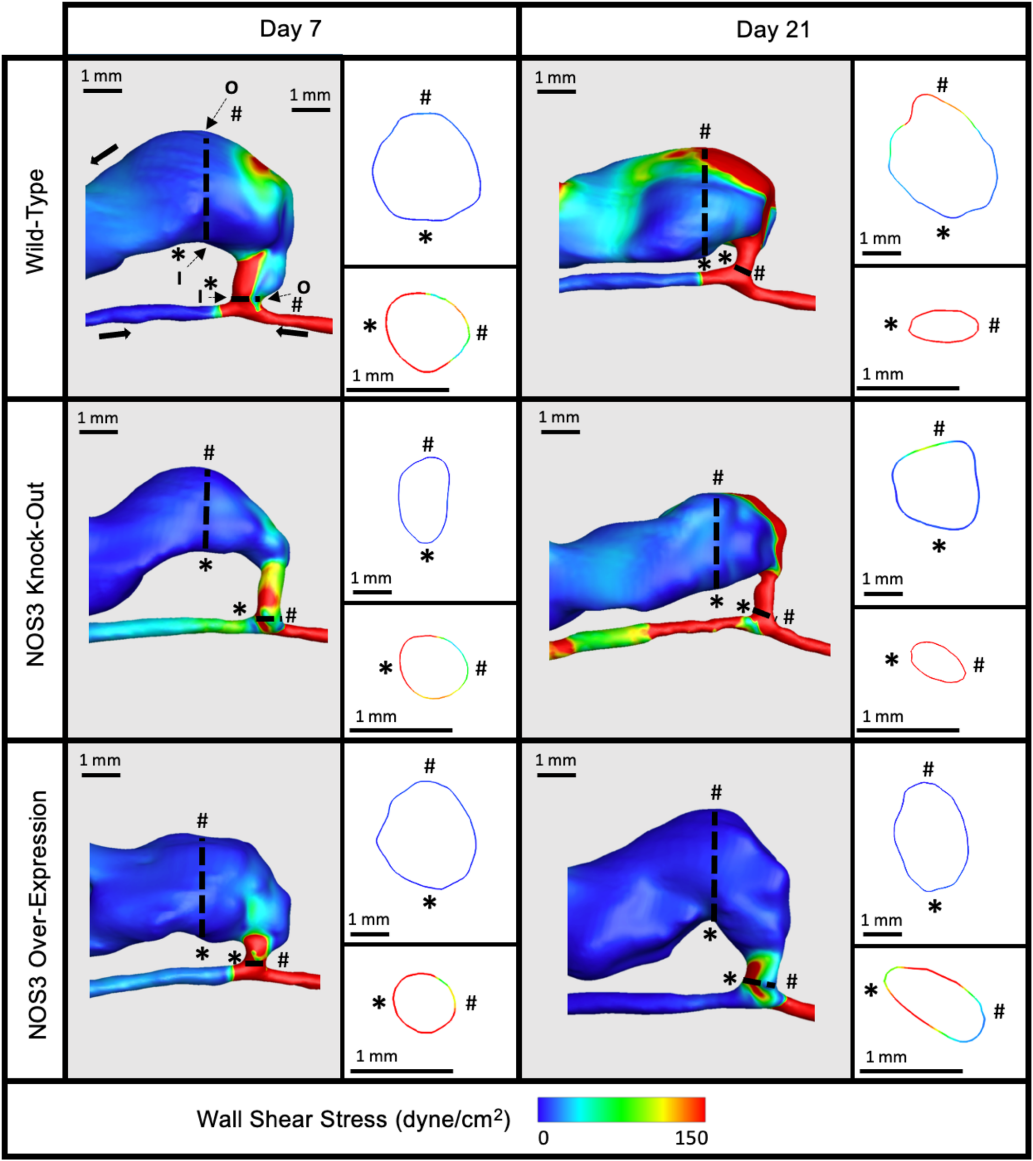
Representative wall shear stress (WSS) contour plot of mouse AVF lumen. Representative WSS contour plot for the three mouse NOS3 strains at 7 and 21 days post-AVF creation at systole. Slices of the AVF lumen WSS at the anastomosis (bottom) and 3 mm into the AVF venous limb (top) are shown next to each plot showing the whole AVF. The WSS color bar applies to all mouse AVFs. The red represents WSS at and above 150 dyne/cm^2^.

**Figure 4:**
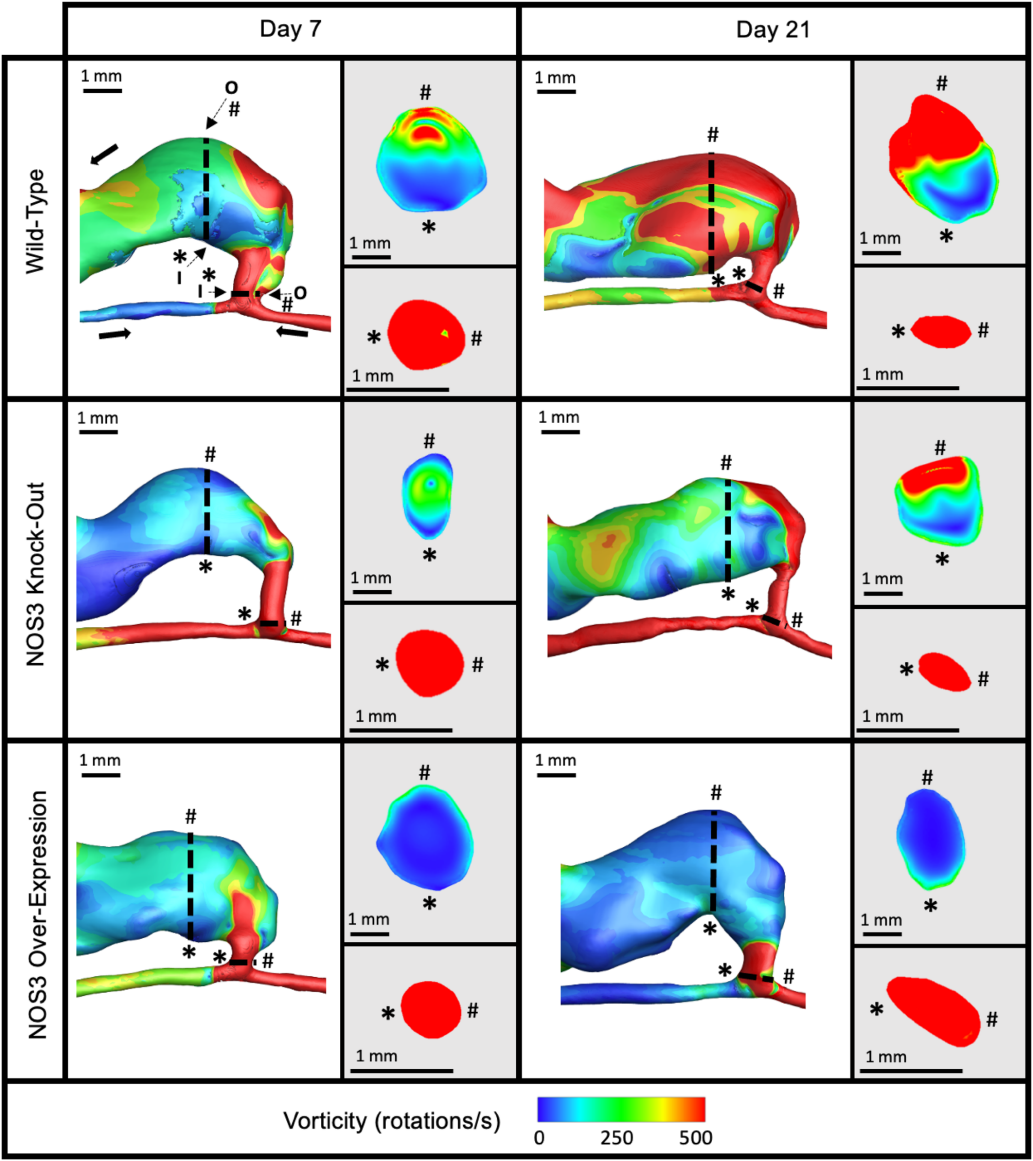
Representative vorticity isosurfaces in mouse AVF lumen. Representative vorticity isosurfaces for the three mouse NOS3 strains at 7 and 21 days post-AVF creation at systole. Slices of AVF lumen velocity at the anastomosis (bottom) and 3 mm into the AVF venous limb (top) are shown next to each plot showing the whole AVF. The vorticity color bar applies to all mouse AVFs. The red represents vorticity at and above 500 rotations/s.

The hemodynamic parameters were reported as velocity streamlines, WSS contour, and vorticity isosurfaces; these are presented with representative mouse AVFs in Figs. 2, 3, and 4, respectively.

Figure 2 shows the velocity streamlines in mouse AVFs for three NOS3 strains at systole. The WT and NOS3 KO mouse AVFs were relatively similar. From day 7 to day 21, aberrant flow developed (i.e., unsmooth velocity streamlines) in the venous limb, and the vein and distal artery velocity increased in WT and NOS3 KO. In the proximal artery, the velocity remained the same for WT from day 7 to day 21 and slightly increased from day 7 to day 21 for NOS3 KO. For the NOS3 OE, the mouse AVF showed an opposite trend over time than the other two strains. From day 7 to day 21, the NOS3 OE mouse AVF decreased in aberrant venous flow (i.e., smoother streamlines), velocity blood flow in the vein, and velocity blood flow in the distal artery (Fig. 2). The proximal artery blood flow velocity stayed the same from day 7 to day 21 for NOS3 OE. A detailed view of the velocity streamlines in the anastomosis region and the venous limb away from the anastomosis is shown in Supplementary Figs. S3 and S4, respectively.

Figure 3 shows the WSS contour in the mouse AVFs for three NOS3 strains at systole. WSS was higher than 150 dyne/cm^2^ in the distal artery from day 7 to day 21 in all three strains (Fig. 3). WSS values in the proximal vein and anastomotic region were similar in WT and NOS3 KO, increasing from day 7 to day 21; however, WSS values in the proximal vein and anastomotic region were lower in NOS3 OE at day 7 compared to WT and NOS3 KO, with a decrease in WSS from day 7 to day 21 (Fig. 3). In the proximal artery, WSS increased from day 7 to day 21 for NOS3 KO, stayed relatively the same for WT, and slightly decreased for NOS3 OE (Fig. 3).

Figure 4 shows the vorticity isosurfaces in the mouse AVFs for three NOS3 strains at systole. The same trends observed in the plots of the velocity streamlines (Fig. 2) and WSS contour (Fig. 3) were also observed in the plot of the vorticity isosurfaces. Specifically, WT and NOS3 KO showed increased vorticity in the proximal vein and the anastomotic region from day 7 to day 21. In the proximal artery, the vorticity increased over time in the WT mouse AVF but stayed relatively higher than 500 rotations/s in the NOS3 KO mouse AVF at each time point. For the NOS3 OE, vorticity decreased throughout the venous limb and proximal artery from day 7 to day 21 (Fig. 4). The distal artery and anastomosis region had a vorticity higher than 500 rotations/s for all three strains at all time points. A detailed view of streamline rods overlayed with the vorticity in each mouse AVF strain is shown in Supplementary Fig. S5.

### Averaged quantitative AVF hemodynamic parameters

**Figure 5:**
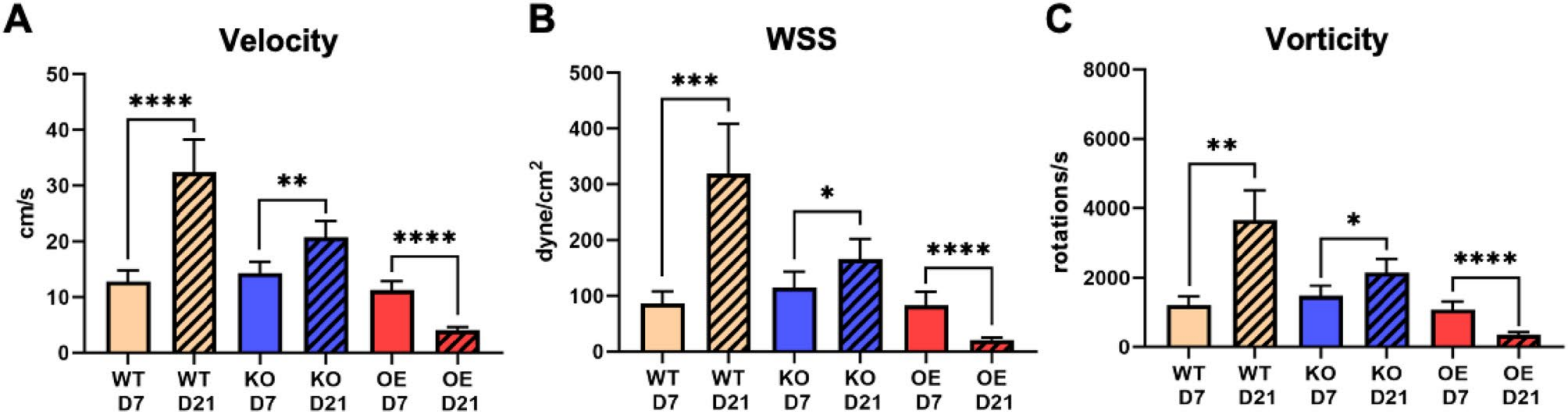
Averaged hemodynamic parameters in mouse AVF proximal vein. The hemodynamic parameters presented are (**A**) velocity, (**B**) wall shear stress (WSS), and (**C**) vorticity. Parameters were averaged 4 mm starting from the anastomosis into the proximal vein. Data are presented as mean ± SEM. An unpaired *t*-test was used to test for statistical differences between groups. *p < 0.05; **p < 0.01; ***p < 0.001; ****p < 0.0001.

**Figure 6:**
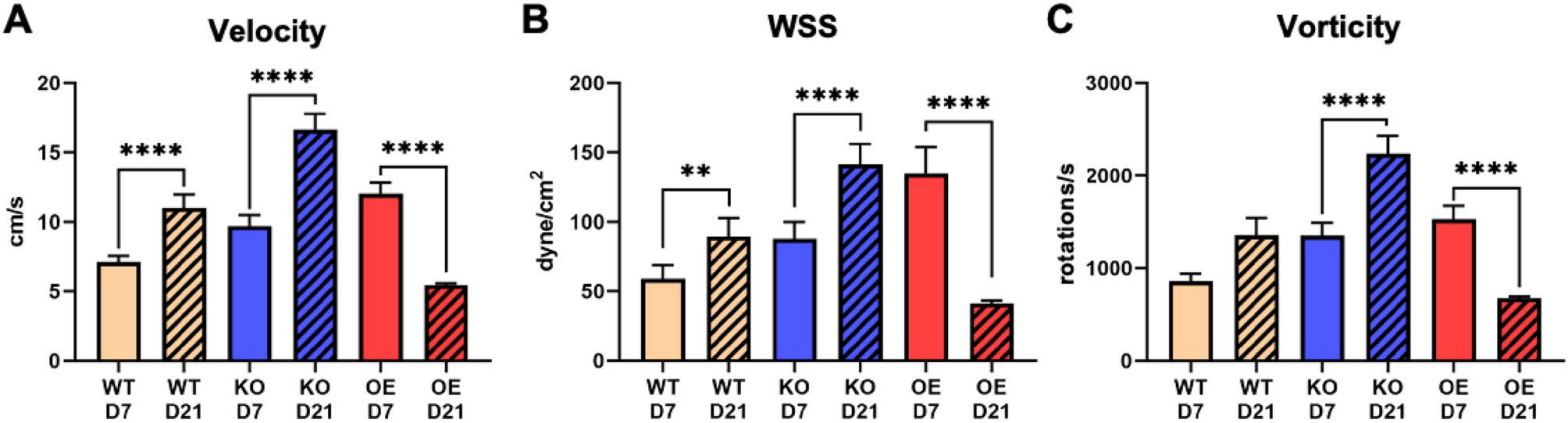
Averaged hemodynamic parameters in mouse AVF proximal artery. The hemodynamic parameters presented are (**A**) velocity, (**B**) wall shear stress (WSS), and (**C**) vorticity. Parameters were averaged 4 mm starting from the anastomosis into the proximal artery. Data are presented as mean ± SEM. An unpaired *t*-test was used to test for statistical differences between groups. **p < 0.01; ****p < 0.0001.

We conducted a detailed quantitative analysis for the proximal vein (Fig. 5) and the proximal artery (Fig. 6) by considering the hemodynamics with 4 mm of each segment starting from the anastomosis. A summary of the hemodynamic parameter results for each condition can be found in Table 1.

**Table 1:** Hemodynamic parameters for proximal vein and proximal artery.

	Velocity (cm/s)		WSS (dyne/cm^2^)		Vorticity (rotations/s)	
	Day 7 (n = 5)	Day 21 (n = 3)	Day 7 (n = 5)	Day 21 (n = 3)	Day 7 (n = 3)	Day 21 (n = 3)
Proximal vein						
WT	12.82 ± 12.84	32.37 ± 38.07	86.96 ± 136	318.7 ± 573.7	1213 ± 1536	3661 ± 5496
KO	14.3 ± 12.92	20.72 ± 19.29	114.92 ± 183.2	165.89 ± 233.8	1479 ± 1855	2141 ± 2549
OE	11.22 ± 10.81	4.09 ± 3.64	83.66 ± 154.5	20.84 ± 30.79	1076 ± 1544	357.1 ± 477.1
Proximal artery						
WT	7.1 ± 2.98	11 ± 6.29	58.84 ± 68.42	89.45 ± 84.79	857.6 ± 532.5	1357 ± 1197
KO	9.7 ± 5.21	16.66 ± 7.27	87.65 ± 80.06	141.3 ± 94.12	1352 ± 911.4	2235 ± 1231
OE	12.04 ± 5.17	5.47 ± 0.74	134.9 ± 120.9	41.31 ± 11.96	1532 ± 903.2	672.8 ± 131.7

Data reported as mean ± standard deviation.

Our results in the proximal vein segment showed that each NOS3 strain exhibited statistically significant temporal changes from day 7 to day 21. The significance varied in *p*-values and is presented as the mean ± standard error of the mean (SEM), as shown in Fig. 5. Quantitative results had similar trends for WT and NOS3 KO, as depicted in Fig. 2. From day 7 to day 21, WT velocity (12.82 ± 2.01 versus 32.37 ± 5.95 cm/s, *p* < 0.0001), WSS (89.96 ± 21.23 versus 318.7 ± 89.6 dyne/cm^2^, *p* < 0.001), and vorticity (1213 ± 239.9 versus 3661 ± 858.3 rotations/s,* p* < 0.01) increased significantly (Fig. 5). A significant increase in velocity (14.3 ± 2.02 versus 20.72 ± 3.01 cm/s, *p* < 0.01), WSS (114.92 ± 28.61 versus 165.89 ± 36.51 dyne/cm^2^, *p* < 0.05), and vorticity (1479 ± 289.8 versus 2141 ± 398.1 rotations/s, *p* < 0.05) was also found for NOS3 KO over time (Fig. 5). In contrast, a significant decrease in velocity (11.22 ± 1.69 versus 4.09 ± 0.57 cm/s, *p* < 0.0001), WSS (83.66 ± 24.12 versus 20.84 ± 4.81 dyne/cm^2^, *p* < 0.0001), and vorticity (1076 ± 241.2 versus 357.1 ± 74.52 rotations/s, *p* < 0.0001) was seen in NOS3 OE from day 7 and day 21 post-AVF creation (Fig. 5). At day 7, the hemodynamic parameters were not statistically significant between WT, NOS3 KO, and NOS3 OE. However, the hemodynamic parameters of WT, NOS3 KO, and NOS3 OE were statistically significant from each other at day 21. WT had a significantly higher velocity, WSS, and vorticity than NOS3 KO and OE, and NOS3 OE had a significantly lower velocity, WSS, and vorticity than WT and NOS3 KO (p < 0.05).

The same as our *venous* analysis, all three NOS3 strains showed significant temporal differences in the *arterial* hemodynamic parameters from day 7 to day 21 (Fig. 6). From day 7 to day 21, WT showed a significant increase in velocity (7.1 ± 0.47 versus 11 ± 0.98 cm/s, *p* < 0.0001) and WSS (58.84 ± 9.88 versus 89.45 ± 13.24 dyne/cm^2^, *p* < 0.01) but not vorticity (857.6 ± 83.17 versus 1357 ± 186.9 rotations/s, *p* = 0.062) (Fig. 6). For NOS3 KO, there was a significant increase in velocity (9.7 ± 0.81 versus 16.66 ± 1.14 cm/s, *p* < 0.0001), WSS (87.65 ± 12.5 versus 141.3 ± 14.7 dyne/cm^2^, *p* < 0.0001), and vorticity (1352 ± 142.3 versus 2235 ± 195.2 rotations/s, *p* < 0.0001) from day 7 to day 21 (Fig. 6). For NOS3 OE, there was a significant decrease in velocity (12.04 ± 0.81 versus 5.47 ± 0.11 cm/s, *p* < 0.0001), WSS (134.9 ± 18.89 versus 41.31 ± 1.87 dyne/cm^2^, *p* < 0.0001), and vorticity (1532 ± 141.1 versus 672.8 ± 20.57 rotations/s, *p* < 0.0001) from day 7 to day 21 (Fig. 6). At day 7, the velocity and WSS for NOS3 KO were significantly higher than WT and NOS3 OE, and the velocity and WSS for NOS3 OE were significantly lower than WT and NOS3 KO (*p* < 0.05). The vorticity at day 7 for NOS3 OE was significantly lower than NOS3 KO and WT. Velocity, WSS, and vorticity at day 21 also differed statistically between each NOS3 strain. NOS3 KO had a significantly higher velocity, WSS, and vorticity than the other strains, while NOS3 OE had a significantly lower velocity, WSS, and vorticity than the other strains (*p* < 0.0001).

### Spatial heterogeneity of AVF vein hemodynamical parameters

**Figure 7:**
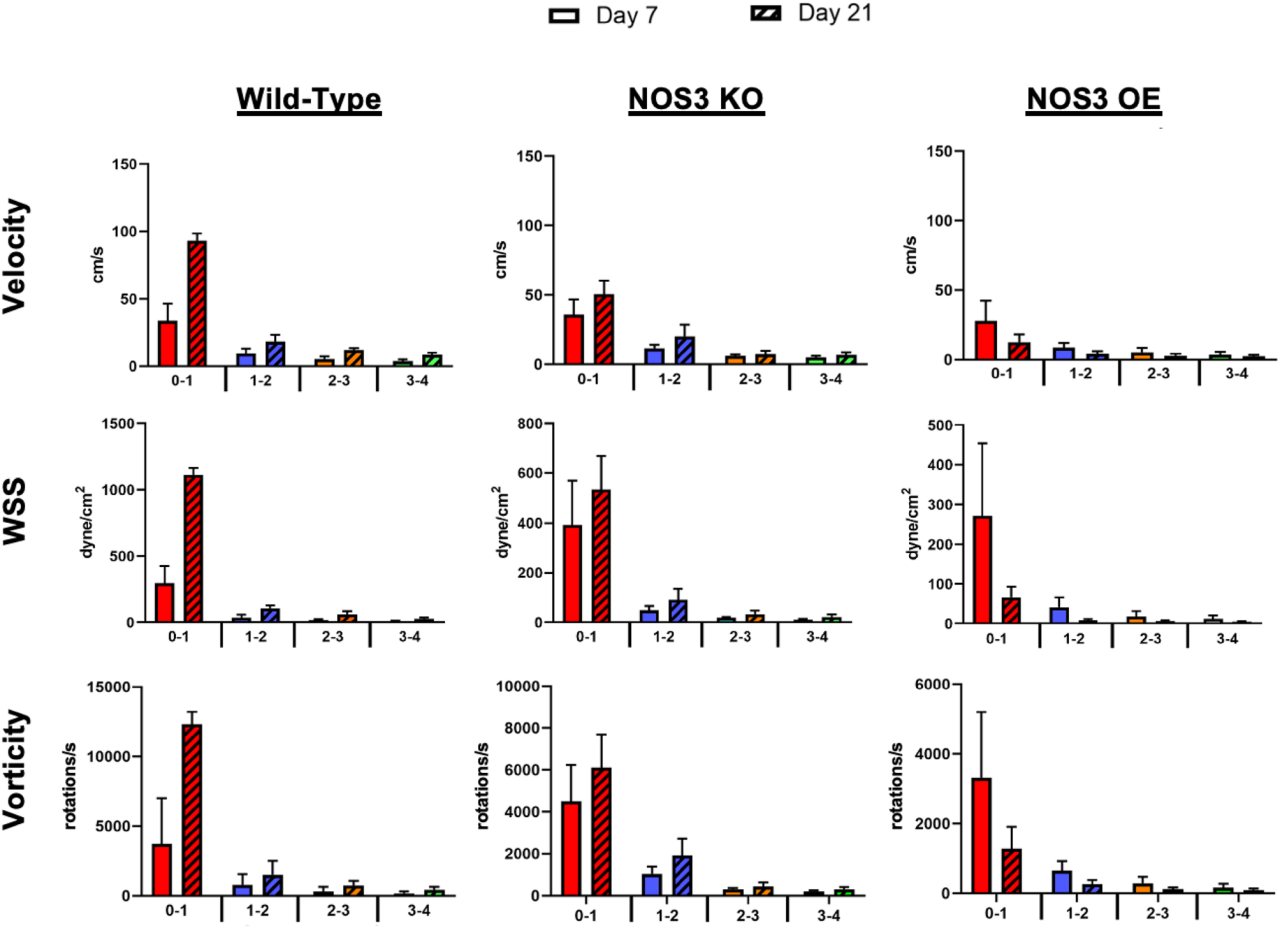
Hemodynamic parameters of WT, NOS3 KO, and NOS3 OE in the proximal vein in 1 mm zones. Velocity, wall shear stress (WSS), and vorticity in the vein in 1 mm zones within 4 mm starting from the anastomosis for WT, NOS3 KO, and NOS3 OE at day 7 (no pattern) and day 21 (patterned) post-AVF creation. Data are presented as mean ± SEM.

The AVF lumen geometry is not uniform and has high spatial heterogeneity. NH has often been observed in the juxta-anastomotic veins of AVFs that fail to mature^17^. Therefore, we performed more granular analysis at and near the anastomosis. Specifically, hemodynamic parameters were analyzed locally in the AVF vein to investigate how the hemodynamic parameters change throughout the vein (Fig. 7). Data was extracted in four zones in the AVF vein, each in a 1-mm increment from the anastomosis, for velocity, WSS, and vorticity (Supplemental Figs. S6, S7, S8, respectively).

The velocity was the highest near the anastomosis in the 0–1 mm zone for all the NOS3 strains (Fig. 7). The temporal velocity changes in each mm of the vein generally followed the same trend of the averaged velocity in the vein over time for each NOS3 strain, as seen in Fig. 5A. From day 7 to day 21, velocity increased significantly for WT in all four zones (*p* < 0.001). The largest increase in velocity for WT occurred in the 0–1 mm zone (175.95% increase, *p* < 0.001). For NOS3 KO, velocity increased significantly for each zone (*p* < 0.01) except for the 1–2 mm zone. NOS3 KO had the largest increase in velocity over time in the 0–1 mm zone (41.36% increase, *p* < 0.0001). For NOS3 OE, a significant decrease in velocity from day 7 to day 21 was found for each 1 mm zone (*p* < 0.001); the largest decrease in velocity was also found in the 0–1 mm zone (51.34% decrease, *p* < 0.001).

WSS was the highest near the anastomosis in the 0–1 mm zone for all three NOS3 strains (Fig. 7). The temporal changes of WSS in each mm of the vein generally followed the same trend of the averaged WSS in the vein over time for each NOS3 strain, as seen in Fig. 5B. From day 7 to day 21, WSS increased significantly for WT in all four zones (*p* < 0.0001), whereas WSS decreased significantly for OE in all four zones (*p* < 0.001). The largest increase in WSS over time for WT was in the 0–1 mm zone (277.42% increase, *p* < 0.0001). The largest decrease in WSS over time for OE was also in the 0–1 mm zone (75.48% decrease, *p* < 0.001). For the NOS3 KO strain, although there was no significant difference in the WSS of the NOS3 KO strain from day 7 to day 21 in the 0–1 mm and 1–2 mm zones (Fig. 7), WSS increased significantly over time in the 2–3 mm (78.22% increase, *p* < 0.05) and 3–4 mm zones (84.34% increase, *p* < 0.0001).

The highest vorticity was found near the anastomosis in the 0–1 mm zone for all three strains (Fig. 7). The vorticity in each mm of the vein relatively followed the same trend of the averaged vorticity in the vein over time for each NOS3 strain, as seen in Fig. 5C. WT increased significantly in vorticity from day 7 to day 21 in all four zones (*p* < 0.001), with the largest increase in the 0–1 mm zone (230.98% increase,* p* < 0.001). For NOS3 OE, vorticity decreased significantly in all four zones (p < 0.01), with the largest decrease in the 0–1 mm zone (61.46% decrease, *p* < 0.01). For NOS3 KO, vorticity increased significantly in each zone (*p* < 0.01) except in the 1–2 mm zone; the largest significant increase in vorticity over time for NOS3 KO was in the 3–4 mm zone (50.69% increase, *p* < 0.0001).

## Discussion

Our present study found that from day 7 to day 21 post-AVF creation, overexpressing NOS3 in a mouse AVF model led to smoother blood flow streamlines, lower WSS, and lower luminal fluid vorticity at day 21, all of which are the desired hemodynamic adaptation. These results build on work our research team has previously conducted^13,14^, and expand to provide a rich data set on the hemodynamics during AVF remodeling in a mouse model. Specifically, our present study is the first to reveal hemodynamics over time within the proximal vein and artery in mouse AVFs and consider the spatial heterogeneity of the un-uniform lumen shape within the vein. This is also the first study to reveal the effect of NOS3 on the natural history of mouse AVF hemodynamics during AVF remodeling. This study further establishes that NOS3 is a critical molecule to driving successful AVF maturation and that the effect of NOS3 can be manipulated in a mouse model to improve the hemodynamics during AVF remodeling.

Previously, our research team conducted a geometric analysis study of our AVF mouse model on day 7 and day 21 post-AVF creation in 21 mice. That study found that the overexpression of NOS3 enhances AVF lumen expansion compared to the WT and NOS3 KO without affecting lumen shape, such as the anastomosis angle and tortuosity of the AVF^14^. Another prior work from our research team conducted an FSI study with our mouse model on day 21 post-AVF creation with a small sample size (*n* = 1 per group), and the value of the hemodynamic parameters averaged over the length of the vein only. That study found that the NOS3 OE mouse had smoother velocity streamlines and lower WSS at day 21 than WT and NOS3 KO, suggesting that NOS3 promotes desired AVF hemodynamics^13^. The hemodynamic results of the present study agree with our previous FSI study using the same mouse model^13^. Importantly, the present study provides a rich dataset on the effect of NOS3 on hemodynamics during AVF remodeling in both the venous and arterial limbs of an AVF, with a granular analysis within the venous limb. Our new results show that from day 7 to day 21 post-AVF creation, the hemodynamics for the WT, NOS3 KO, and NOS3 OE mouse AVFs were all undergoing hemodynamic remodeling. The WT and NOS3 KO mouse AVFs significantly increased in blood flow velocity, WSS, and vorticity from day 7 to day 21 post-AVF creation. In contrast, the NOS3 OE mouse AVFs had decreased blood flow velocity, WSS, and vorticity over time.

In a majority of the mouse AVFs, we saw a retrograde flow in the distal artery, with flow entering the proximal vein coming from the distal artery in addition to from the proximal artery. This is likely caused by a low-resistance AVF circus with low pressure, leading to the reversal of the blood flow in the distal artery. Retrograde flow in the distal artery is commonly seen in human AVFs suffering from steal syndrome^18,19^.

An innovation of this study is that our study is the first to show the effect of NOS3 on the WSS over time in a *mouse* model. Previous studies have associated the reduction of WSS over time with favored remodeling in human AVFs^20^ and porcine AVFs^21,22^. The reduction of WSS over time has been linked to an increase in lumen area, an increase in flow rate, and decreased intima medial thickening in the arterial and venous segments of the porcine AVF^21–23^. Our results suggest that WSS was attempting to return to baseline values, and velocity streamlines were smoother and less aberrant in the NOS3 OE mouse AVFs compared to those in the WT and NOS3 KO. This is supported by the decrease in WSS and vorticity for the NOS3 OE mouse AVFs from day 7 to day 21 post-AVF creation. The finding of low vorticity (i.e., less disturbed flow) in the NOS3 OE mouse AVFs is also significant, as previous studies have found that disturbed flow is correlated with the development of NH and subsequent stenosis after AVF creation^24,25^. Additionally, a prior multi-center, prospective human study found strong clinical evidence that a high OSI was negatively associated with AVF lumen expansion^20^. Therefore, our results from the mouse AVF model indicate that the overexpression of NOS3 OE leads to characteristics of favored remodeling that would lead to successful AVF maturation, as seen in humans.

The increase in velocity, WSS, and vorticity over time observed in the WT and NOS3 KO mouse AVFs suggests the presence of higher aberrant flow, indicative of unfavorable remodeling and an increased rise of AVF maturation failure^23^. The increase in the WSS or vorticity of WT and NOS3 KO during the remodeling period from day 7 to day 21 may be due to stenosis or the development of NH. This agrees with a previous histology study on our mouse model that showed venous NH development in NOS3 KO and WT mouse AVFs, while the NOS3 OE mouse AVF showed restricted NH formation^26^.

Another innovation of this study is the detailed analysis of the effect of NOS3 on the hemodynamics locally within the vein. We found that the hemodynamics for all the mouse AVFs were higher near the anastomosis and decreased going downstream of the vein. This observation is consistent with prior literature on hemodynamics in pig and human AVFs^21,27^ as well as our research team’s previous rat study^16^. The change in blood pressure causes higher hemodynamics near the anastomosis as blood goes from a high-pressure environment in the artery to a low-pressure environment in the vein. Studies have shown that stenosis in AVFs most often occurs in the anastomotic and juxta anastomotic regions, though it also occurs in the vein further downstream^28–31^. The incidence of inflow stenosis (i.e., stenosis at the anastomosis or juxta anastomotic region) in a multi-center, prospective human study found that access inflow stenosis occurred in 40% of the AVF cases referred to interventional facilities with clinical evidence of venous stenosis or thrombosis^29^. Another study also reported that 45% of patients evaluated for early fistula failure were found to have juxta-anastomotic venous stenosis^28^. This shows that the hemodynamics near the anastomotic region of the vein is critical to study.

The limitations of this study include the mouse-to-mouse variation between day 7 and day 21 post-AVF creation and pixel wrapping in the cine-phase MRI images. We did not conduct the longitudinal study because we did not want to expose the mice to repeated doses of anesthesia. The pixel wrapping was accounted for by excluding as much pixel wrapping as possible on the cine-phase MRI in lumen selection. There are also some tools to correct pixel wrapping in another program called Segment, but for this study, we used ImageJ. Another limitation is the absence of hemodynamic analysis before day 7 post-AVF creation. Past studies have found that the blood flow changes drastically within 1 day after AVF creation^20,32^. Future studies could provide valuable insights into the changes occurring in the AVF from creation to remodeling by including an additional time point before day 7 post-AVF creation. An additional future direction to take is to investigate the hemodynamics in terms of helicity. The beneficial role of helical flow in the human cardiovascular system is well known^33^. Several studies have reported that helical flow may suppress flow disturbances that lead to disturbed shear, thus reducing the onset of NH^34–36^. Future studies are warranted to assess the effects of NOS3 on helicity-based hemodynamic parameters within the mouse AVF over time. Our previous FSI study used a two-element R–C Windkessel model for a boundary condition on the distal artery^13^. Future CFD studies can apply this technique as well.

In conclusion, this study agrees with our previous research while expanding and providing a rich hemodynamic data set regarding the effect of NOS3 on AVF remodeling at two time points. Compared to WT and NOS3 KO, the overexpression of NOS3 leads to favorable AVF remodeling, smoother blood streamlines, lower WSS, and lower blood vorticity over time. These results are the first to show the effect of NOS3 on the WSS over time and the hemodynamics locally during AVF remodeling over time. Future studies can investigate enhancing the NOS3 pathway to improve AVF development and remodeling.

### Supplementary Information

Supplementary Figures.

## Data Availability

The datasets generated during and/or analyzed during the current study are available from the corresponding author upon reasonable request.
