# Supplementary material for: Effects of endothelial nitric oxide synthase on mouse arteriovenous fistula hemodynamics
Source: Sci Rep. 2023 Dec 20;13:22786. doi: 10.1038/s41598-023-49573-5 (PMC10733286; doi:10.1038/s41598-023-49573-5)
Supplement: Supplementary file 1 — Supplementary Figures. [file 41598_2023_49573_MOESM1_ESM.docx]

**SUPPLEMENTARY FIGURES**

**Effects of endothelial nitric oxide synthase on mouse arteriovenous fistula hemodynamics**

Shelly Baltazar^1,2^, Hannah Northrup^2^, Joshua Chang^2^, Maheshika Somarathna^3^, Tatyana Isayeva Waldrop^3^, Timmy Lee^3,4^, Yan-Ting Shiu^2,5^

Affiliation:
^1^Department of Biomedical Engineering, University of Utah, Salt Lake City, UT

^2^Division of Nephrology and Hypertension, Department of Internal Medicine, University of Utah, Salt Lake City, UT

^3^Division of Nephrology, Department of Medicine, University of Alabama at Birmingham, AL

^4^Veterans Affairs Medical Center, Birmingham, AL

^5^Veterans Affairs Medical Center, Salt Lake City, UT


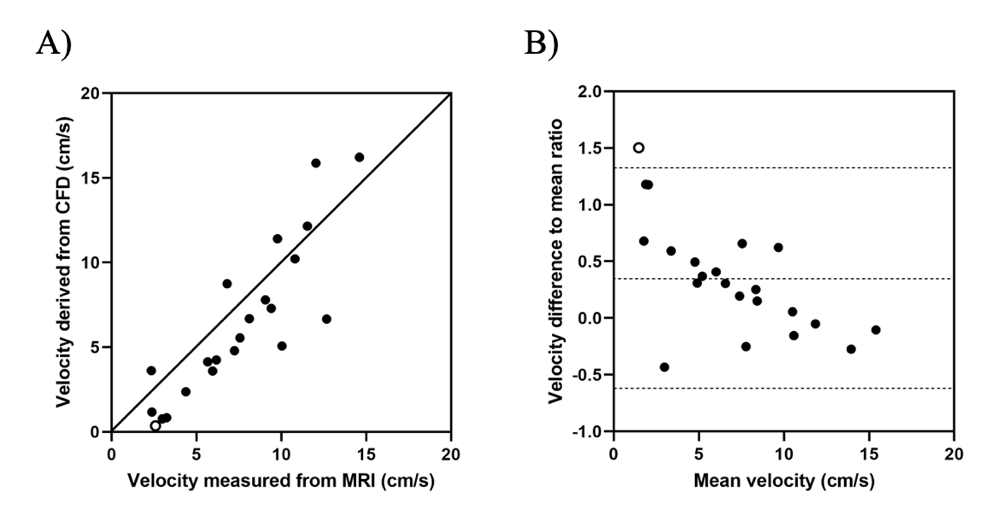


**Supplementary Figure** **S1. Comparison of averaged proximal vein velocity by MRI and CFD.** A) Agreement plot of velocity in the proximal vein derived from CFD and measured by MRI. The diagonal line represents equal MRI-measured and CFD-derived velocities. The outlier identified in (B) is shown as an open circle in (A). B) Bland-Altman plot used to identify outliers. The x-axis was the mean velocity by MRI & CFD, and the y-axis was the MRI-to-CFD velocity difference to mean ratio. The center dashed line represents the mean ratio, and the top and bottom dashed lines represent the mean ratio ± 1.96 x standard deviation of the ratio. One outlier (open circle) was found.


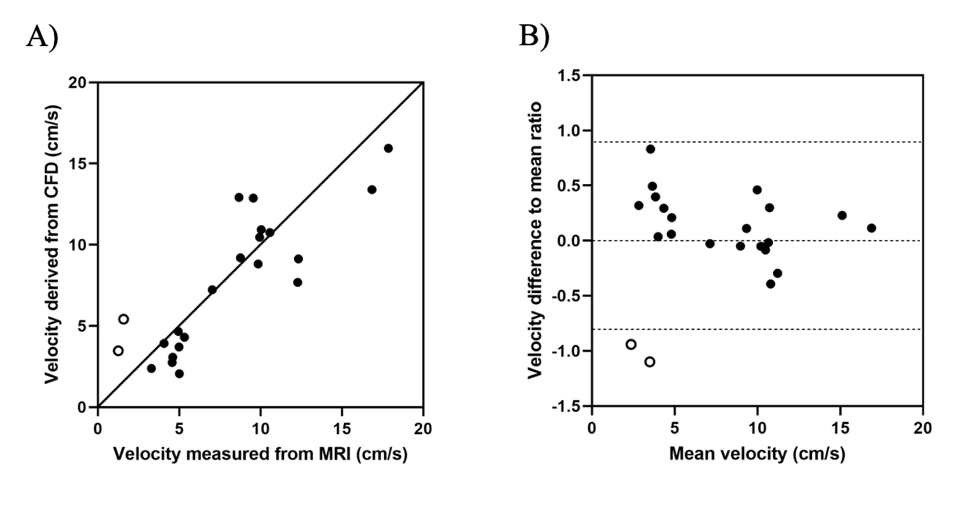


**Supplementary Figure** **S2. Comparison of averaged proximal artery velocity by MRI and CFD.** A) Agreement plot of velocity in the proximal artery derived from CFD and measured by MRI. The diagonal line represents equal MRI-measured and CFD-derived velocities. The outliers identified in (B) are shown as open circles in (A). B) Bland-Altman plot used to identify outliers. The x-axis was the mean velocity by MRI & CFD, and the y-axis was the MRI-to-CFD velocity difference to mean ratio. The center dashed line represents the mean ratio, and the top and bottom dashed lines represent the mean ratio ± 1.96 x standard deviation of the ratio. Two outliers (open circles) were found.


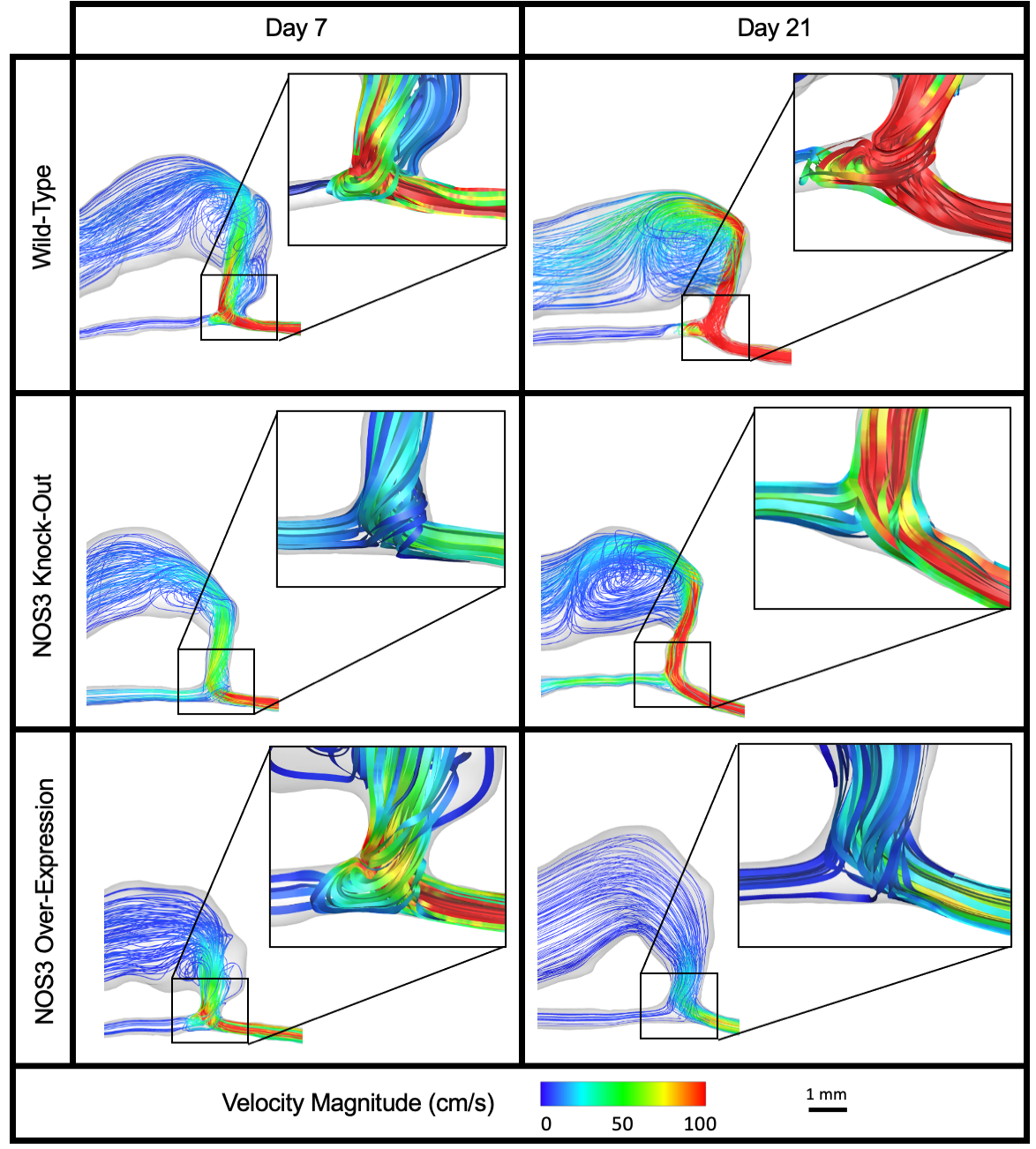


**Supplementary Figure S3. Velocity streamlines with a more detailed view of the anastomotic region.** Representative velocity streamline color plot for the three mouse NOS3 strains at 7 and 21 days post-AVF creation at systole with a detailed view of the anastomosis with velocity ribbons. The velocity color bar and scale bar apply to all mouse AVFs.


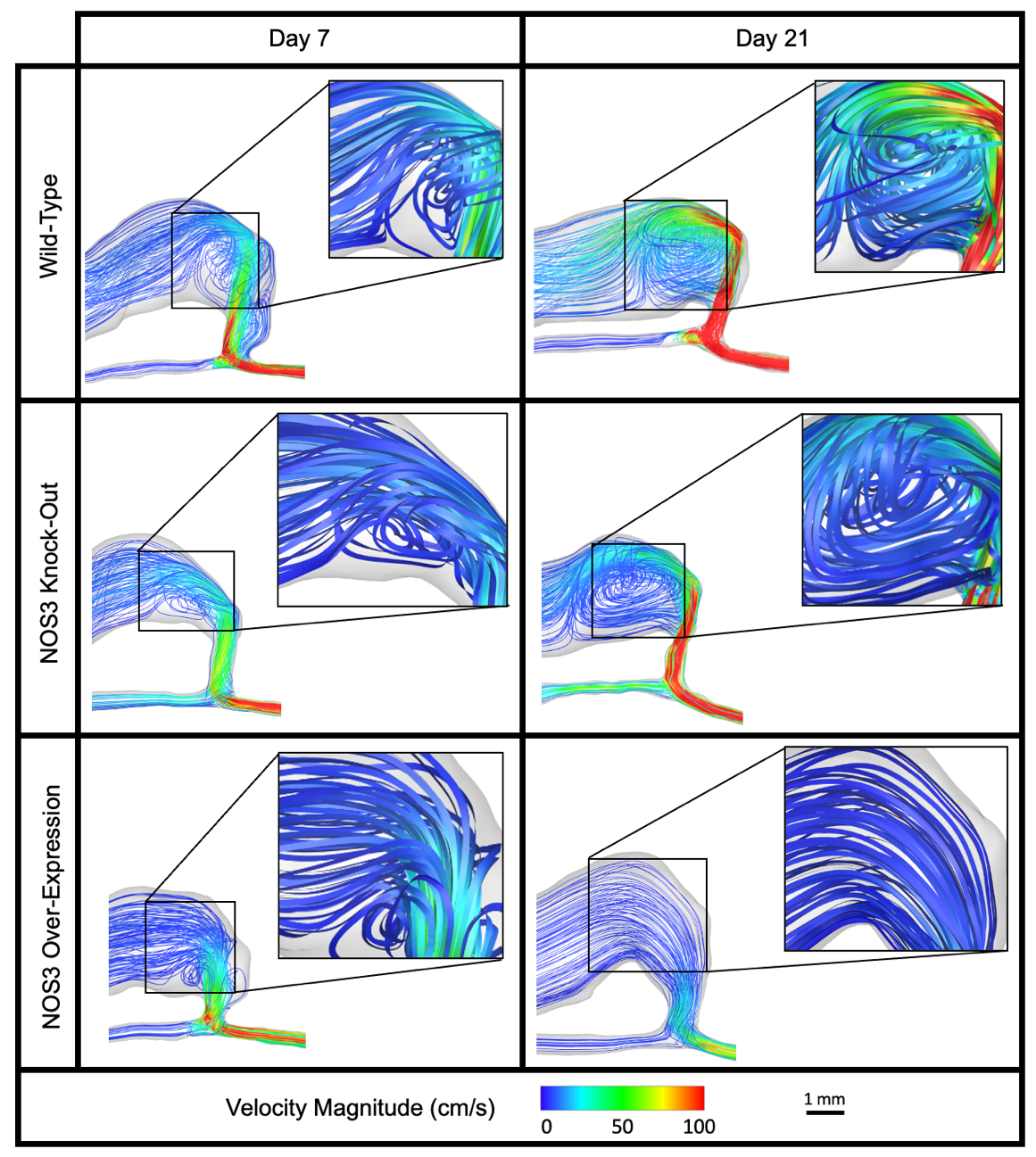


**Supplementary Figure S4. Velocity streamlines with a more detailed view of the venous limb.** Representative velocity streamline color plot for the three mouse NOS3 strains at 7 and 21 days post-AVF creation at systole with a detailed view of the proximal vein with velocity ribbons. The velocity color bar and scale bar apply to all mouse AVFs.


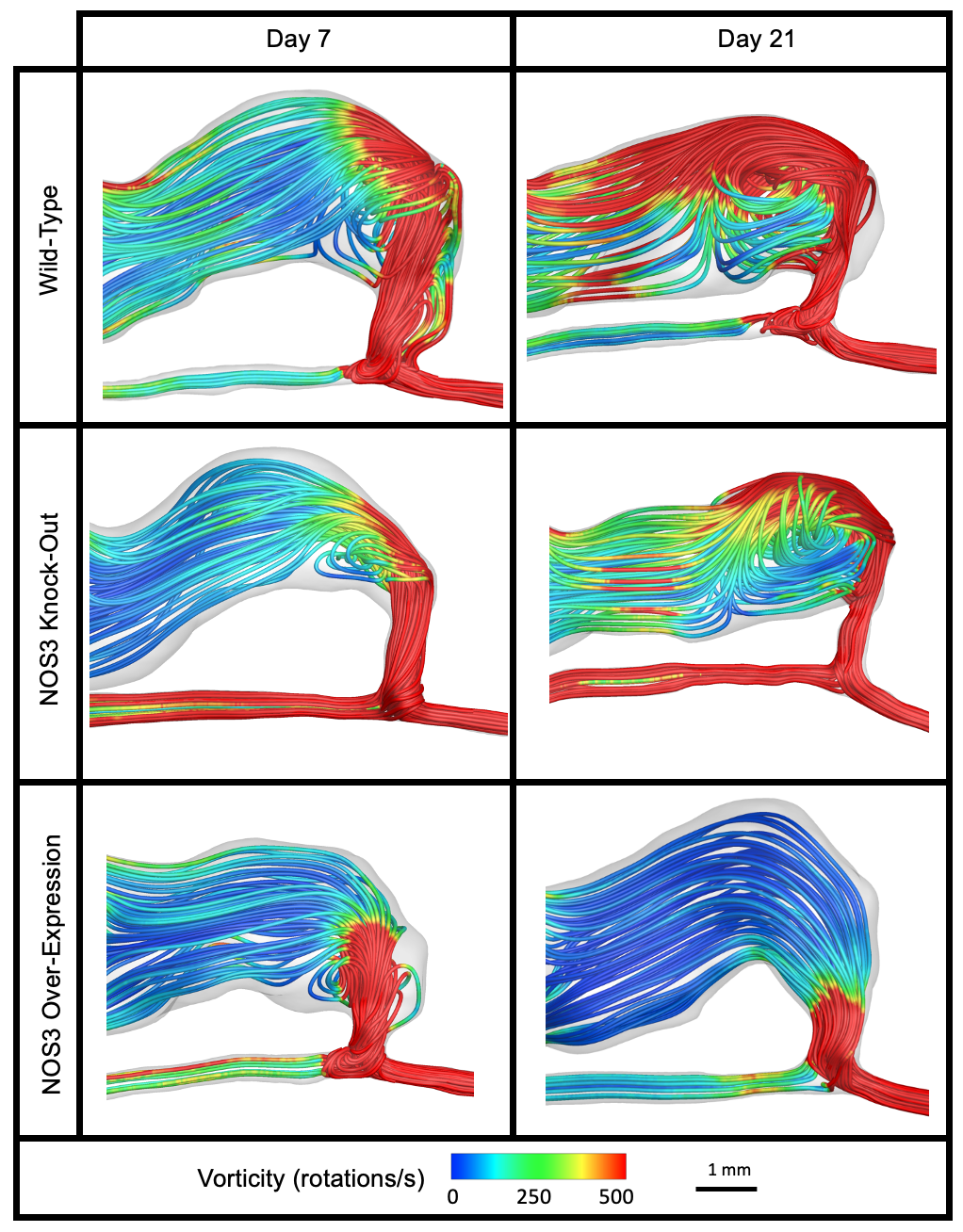


**Supplementary Figure S5. Streamline rods contoured with the vorticity.** Representative streamline rods contoured with the vorticity color plot for the three mouse NOS3 strains at 7 and 21 days post-AVF creation at systole. The vorticity color bar and scale bar apply to all mouse AVFs.


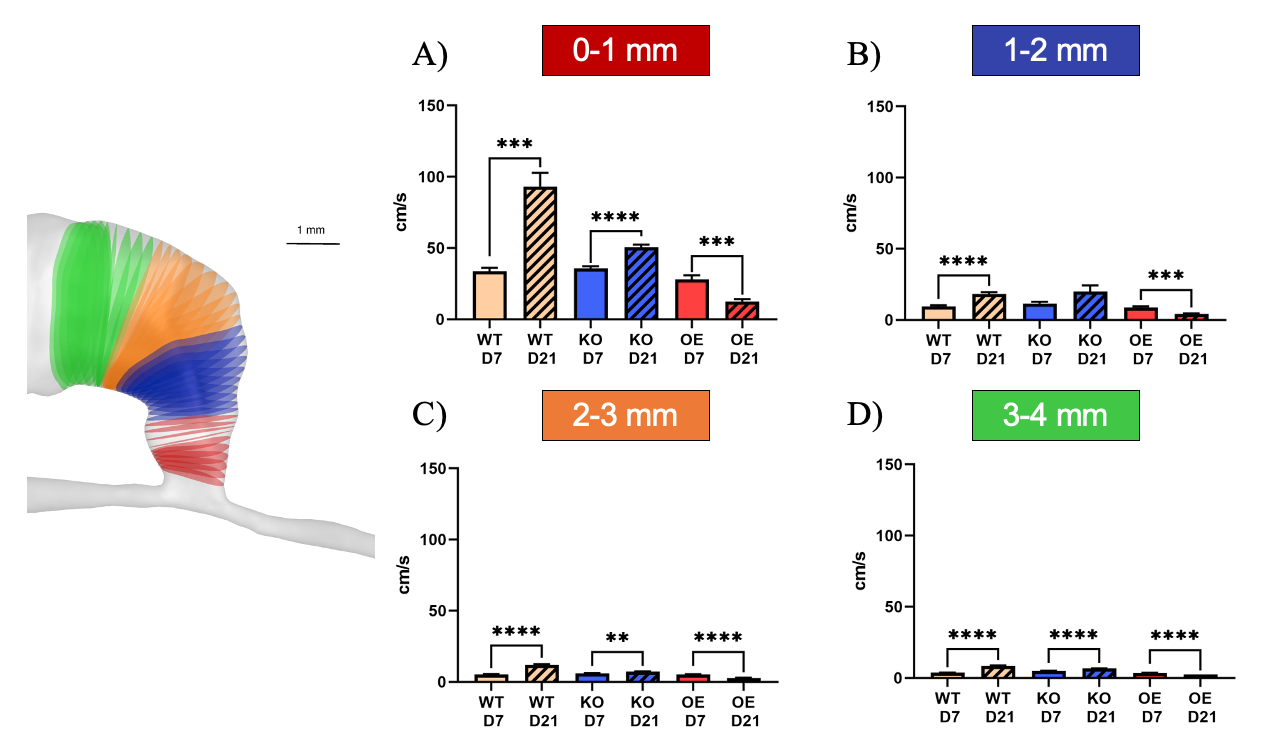


**Supplementary Figure** **S6. Vein velocity in 1-mm zones.** The panels show the average velocity in each zone: A) 0-1 mm, B) 1-2 mm, C) 2-3 mm, and D) 3-4 mm. The left shows the representative colors for each 1-mm zone. Slices were extracted every 0.1 mm, 10 slices per zone. Data presented as mean ± SEM. An unpaired *t*-test was used to test for statistical differences between groups. **: *p*<0.01; ***: *p*<0.001; ****: *p*<0.0001.


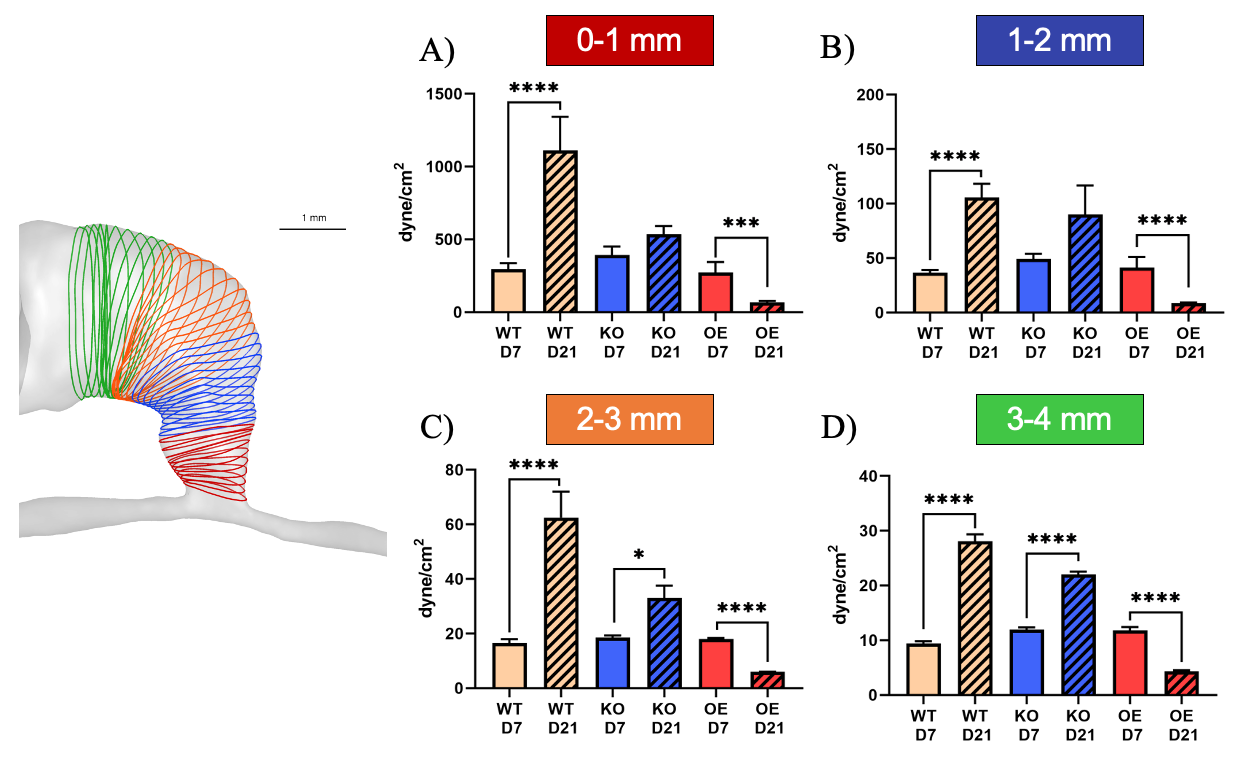


**Supplementary Figure** **S7. Vein WSS in 1-mm zones.** The panels show the average WSS in each zone: A) 0-1 mm, B) 1-2 mm, C) 2-3 mm, and D) 3-4 mm. The left shows the representative colors for each 1-mm zone. Slices were extracted every 0.1 mm, 10 slices per zone. Data presented as mean ± SEM. An unpaired *t*-test was used to test for statistical differences between groups. *:p<0.05; ***: *p*<0.001; ****: *p*<0.0001.


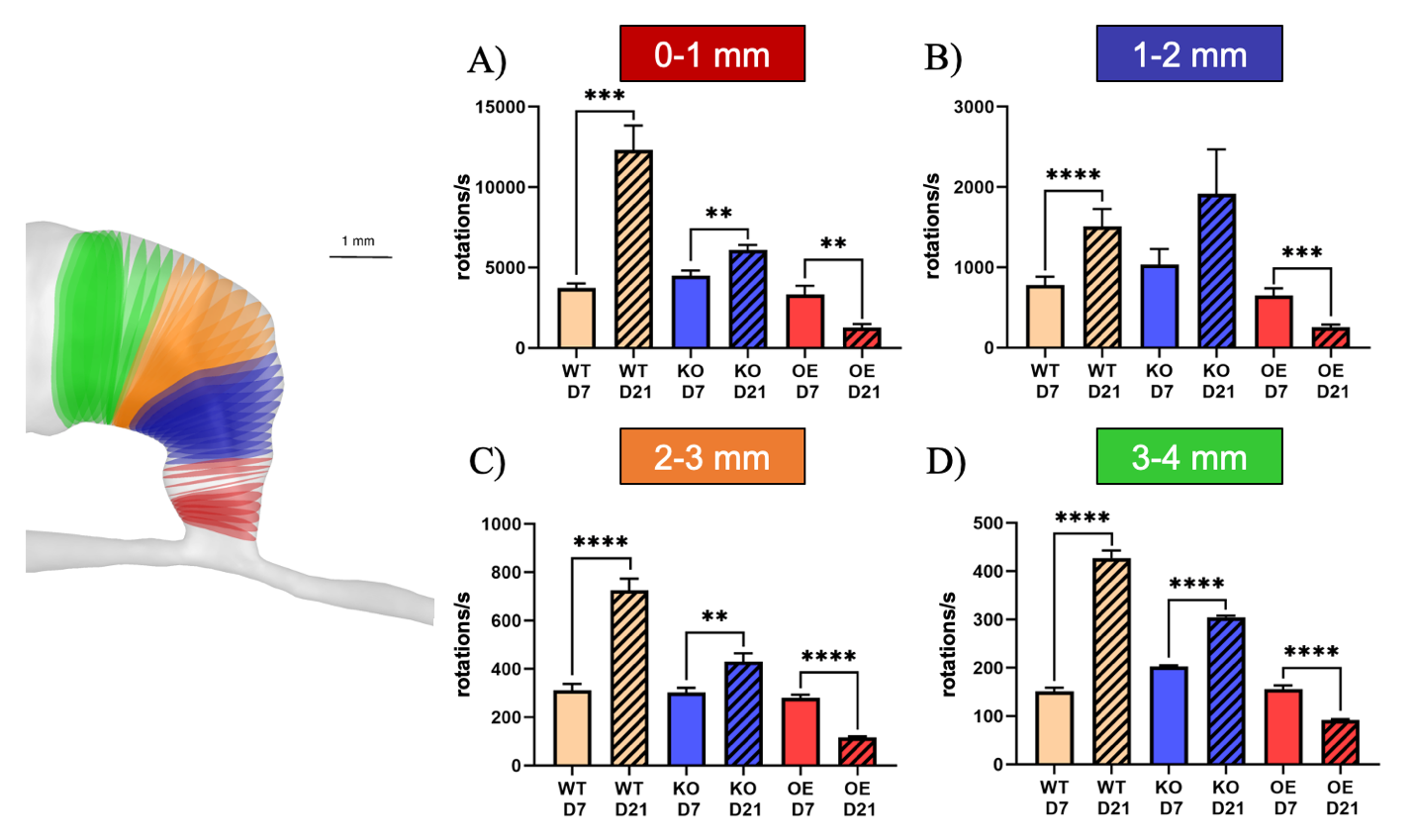


**Supplementary** **Figure S8. Vein vorticity in 1-mm zones.** The panels show the average vorticity in each zone: A) 0-1 mm, B) 1-2 mm, C) 2-3 mm, and D) 3-4 mm. The left shows the representative colors for each 1-mm zone. Slices were extracted every 0.1 mm, 10 slices per zone. Data presented as mean ± SEM. An unpaired *t*-test was used to test for statistical differences between groups. **: *p*<0.01; ***: *p*<0.001; ****: *p*<0.0001.
